# Animal Reservoir Hosts and Fish-borne Zoonotic Trematode Infections on Fish Farms, Vietnam

**DOI:** 10.3201/eid1504.081147

**Published:** 2009-04

**Authors:** Nguyen Thi Lan Anh, Nguyen Thi Phuong, K. Darwin Murrell, Maria Vang Johansen, Anders Dalsgaard, Luong To Thu, Tran Thi Kim Chi, Stig Milan Thamsborg

**Affiliations:** National Institute of Veterinary Research, Hanoi, Vietnam (N.T.L. Anh, N.T. Phuong, L.T. Thu); University of Copenhagen, Copenhagen, Denmark (N.T.L. Anh, D. Murrell, M. Vang Johansen, A. Dalsgaard, S.M. Thamsborg); Research Institute for Aquaculture I, Tu Son, Bắc Ninh, Vietnam (T.T.K. Chi)

**Keywords:** Zoonosis, fish-borne trematodes, parasites, reservoir hosts, fish, aquaculture, domestic animals, Vietnam, research

## Abstract

Fish-borne zoonotic trematodes (FZT) pose a risk to human food safety and health and may cause substantial economic losses in the aquaculture industry. In Nghe An Province, Vietnam, low prevalence of FZT for fish farmers but high prevalence for fish indicate that reservoir hosts other than humans may play a role in sustaining transmission. To determine whether domestic animals may be reservoir hosts, we assessed prevalence and species composition of FZT infections in dogs, cats, and pigs in a fish-farming community in Vietnam. Feces from 35 cats, 80 dogs, and 114 pigs contained small trematode eggs at 48.6%, 35.0%, and 14.4%, respectively; 7 species of adult FZT were recovered from these hosts. These results, combined with data from previous investigations in this community, imply that domestic animals serve as reservoir hosts for FZT and therefore must be included in any control programs to prevent FZT infection in humans.

## Abstract 2

Cats, dogs, and pigs may be reservoir hosts.

In Asia, fish-borne zoonotic trematodes (FZT), including liver and intestinal flukes, are widely reported (*1*–*3*). FZT not only pose risks to food safety and human health but also may cause substantial economic losses in the aquaculture industry, resulting from to restrictions on exports and reduced consumer demand because of food safety concerns (*4*). A range of mammals and birds serve as definitive hosts for FZT (*2*). Although information on infection levels and species distribution in humans and fish is becoming increasingly available, similar information for reservoir hosts such as wild and domestic animals and fish-eating birds is scarce (*1*).

Recent studies conducted in Nghe An Province, a major area for freshwater aquaculture in Vietnam, found prevalence of FZT in humans to be low (0.6%) and prevalence in fish from farms to be high (>35%) (*5*,*6*). These findings suggest that reservoir hosts other than humans play a major role in sustaining transmission of FZT in this community. We therefore investigated the role of the domestic animals on these fish farms. We determined prevalence and species composition of FZT infections in dogs, cats, and pigs in the community and analyzed potential risk factors for the transmission of FZT to animals and animals’ role in sustaining FZT infections in cultured fish.

## Materials and Methods

### Study Design, Sampling, and Laboratory Analysis

**Figure 1:**
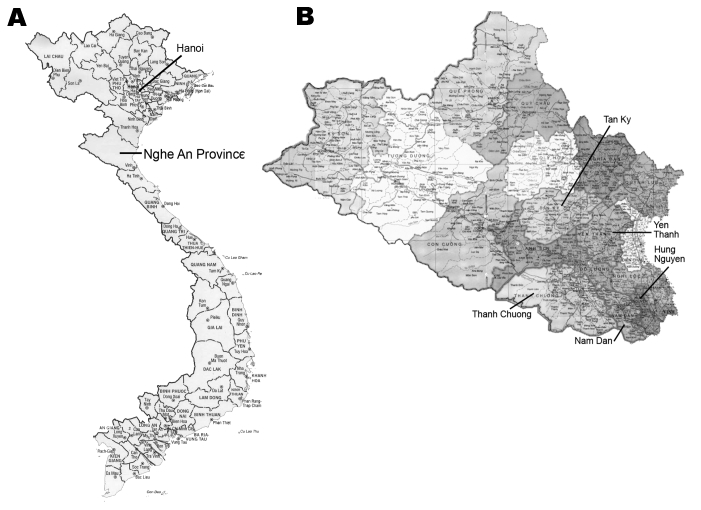
A) Location of Nghe An Province in northern Vietnam. B) Location of the 5 selected districts from which households were selected for investigation of fish-borne zoonotic trematodes in domestic animals.

The study was conducted in November 2005 in Nghe An Province, northern Vietnam (Figure 1), in fish-farming households previously investigated for human and fish FZT infections (*5*,*6*). From a total of 1,281 households, 50 were randomly selected in proportion to farm numbers in 5 districts: Hung Nguyen (n = 8), Nam Dan (n = 15), Yen Thanh (n = 9), Thanh Chuong (n = 10), and Tan Ky (n = 8). Another fish farm previously found to have cases of FZT in humans (*5*) was included, yielding a total of 51 fish-farming households in the study. Before the study, all farmers were informed about the study (objectives, risks, rights, and benefits) and asked for consent. Permission to conduct the study was obtained from National Institute of Veterinary Research.

Fecal samples were collected from every animal in the selected households: 80 dogs, 35 cats, and 114 pigs. Animals that were <2 months of age or pregnant were excluded. Fecal samples were collected from the rectums of dogs and pigs and from the cages of cats that had been confined overnight. Samples were stored in coolers and transferred to the laboratory, where 1–2 mL of 10% formalin was added and the samples were kept refrigerated until examination within 6 weeks. A standard questionnaire was used to interview each animal’s owner about the behavior of the animals and about animal husbandry practices relevant to transmission of FZT.

Fecal samples (5 g each) were examined by a combined filtration, sedimentation, and centrifugation method described by Willingham et al. (*7*) and Lan Anh et al. (*8*). For each sample, trematode eggs were counted 3 times, the sum of which was equivalent to eggs per gram (epg). All eggs <50 µm were designated “small trematode eggs” (*3*). Data on prevalence and species of metacercaria in fish were obtained from Chi et al. (*6*). Human prevalence data, collected according to the Kato-Katz method, were obtained from Olsen et al. (*5*).

### Worm Recovery and Identification

Among the small trematode egg–positive animals, 27 dogs, 18 cats, and 5 pigs were randomly selected for necropsy and adult worm recovery from the liver and small intestine. The animals were housed and handled in accordance with national standards of experimental animal care. Dogs and pigs were anesthetized by intramuscular injection of xylazine and subsequently killed by an intravenous overdose of ketamine (Troy Laboratories Pty.Td, Smithfield, New South Wales, Australia). Cats were killed by an intramuscular overdose of ketamine. The livers were removed and cut open along the main tributaries of the biliary duct, and trematodes were collected and placed in a Petri dish with saline. The liver was subsequently cut into small, thin pieces and placed in saline solution for 10 min, after which the liver tissue was crushed and worms were isolated by filtration of the solution through a tea strainer. The contents of the small intestines were flushed into a bucket by tap water, then filtered through a tea strainer and sieve (mesh size 400 µm). The sediment remaining on the sieve was washed into a Petri dish and examined for intestinal flukes under a stereomicroscope. After 30 min, the sediment in the wash water was also examined for flukes. To recover the remaining flukes, we cut the intestines into small pieces and placed them in a bucket with warm saline (90°C) for 1 h. The bucket fluid was poured into conical flasks and allowed to settle for 30 min before the final sediment was examined in a Petri dish under a stereomicroscope. All isolated flukes were collected by pipette and preserved in saline (90°C) before being pooled in 1 flask and counted. Worms were preserved with 5% formalin in Eppendorf tubes; when high worm loads were isolated, a subsample was preserved in 70% ethanol for later analysis by PCR. As many as 40 formalin-preserved flukes per animal were stained, mounted on slides, and identified to species level according to published taxonomic references (*9*,*10*).

### Relative Transmission Index

We determined total daily eggs excreted (TDEE) for each animal species and for humans by multiplying 4 factors: number of animals and humans in the districts, FZT prevalence for animals and humans, mean epg in feces, and amount of feces excreted per day. A relative transmission index (RTI) was used to assess the potential contribution of animals and humans to FZT transmission. RTI was defined as the proportion of the total daily trematode egg excretion produced by each species and calculated by using the following formula:

RTI = TDEE for each species × 100/TDEE for all species

The estimated amounts of feces defecated daily (mean ± SD) were obtained from Wang et al.: humans 160 ± 58 g, dogs 99 ± 19 g, cats 20 ± 19 g, and pigs 1,516 ± 196 g (*11*)*.* Data on number of persons in the 5 districts were collected from the Vietnam Administrative Atlas (*12*), and data on numbers of domestic animals were collected from local veterinary centers in the districts. Data on prevalence and intensity of the eggs from humans in the same study areas were collected from Olsen at al. (*5*) and from Annette Olsen (pers. comm.), respectively*.*

### Data Analysis

Data from parasitologic examinations were combined with information collected from questionnaires administered to the animals’ owners, recorded on an Excel spreadsheet (Microsoft, Redmond, WA, USA), and transferred to SAS version 9.1 (SAS Institute, Inc., Cary, NC, USA) for statistical analysis. The outcome variable was FZT infection status of the animals (yes/no). Explanatory variables obtained from the questionnaire were sex of animal (male/female); age group (<12 months, >12 months); district; animal species (dogs, cats, pigs); free roaming of the animal (never, sometimes, always); farmer fed raw fish to animal (never, sometimes); farmer observed the animal eating raw fish (never, sometimes); farmer observed the animal catching fish from canal or river (yes/no); farmer’s practice of feeding dead fish from pond to the animals (yes/no); deworming of the animals (never, one time when animals were young, sometimes if animal was ill, regularly [2 times/year]); place where animals defecate (garden, stable, wherever, on the fish pond bank, in the kitchen with straw); farmer’s use of animal feces (rice field, vegetable garden, fish pond, wherever, rice field and fish pond); and composting of feces (never, sometime, always).

For risk factor analysis, univariable and multivariable logistic regression analyses were conducted for 2 subsets of data: all animals and each animal species. Univariable analysis was performed to assess possible risk factors for FZT infection; multivariable logistic regression was used to evaluate the effect of risk factors when adjusting for the effect of other risk factors. Risk factors with p<0.25 in univariable analysis were included in the initial multivariable models. Backward elimination was used to include only risk factors with p<0.05 in the resulting model. Interaction and possible confounding were checked. Where interaction was found, it was included in the resulting model. Goodness-of-fit was checked by the Hosmer-Lemeshow strategy. Comparisons of prevalence of the infections between animal species were performed by using the Fisher exact test.

Data from parasitologic examination of fish in the same households (*6*) were used to assign codes to households: code 1 (positive) if any fish had FZT metacercariae and code 0 if all fish examined were negative. The Fisher exact test was used to analyze relationships at the household level between the infections in fish and in domestic animals together or between infections in fish and each animal species.

## Results

### Prevalence and Species Composition of Small Trematodes

The prevalence of small trematode infections, determined by egg counts in fecal samples from 229 animals of 51 households, was 35.0% for dogs, 48.6% for cats, and 14.4% for pigs (Table 1). Prevalence of infection was higher for cats and dogs than for pigs (p<0.0001), but no significant difference was found between prevalence of infections for dogs and cats (p = 0.21). Intensity (small trematode egg counts; mean ± SD) was highest for cats (66 ± 129 epg), intermediate for dogs (25 ± 73 epg), and lowest for pigs (4 ± 18 epg).

**Table 1:** Small trematode infections in domestic animals, Nghe An Province, Vietnam, November 2005*

Sample source	No. animals sampled	Infection prevalence, %†	Egg intensity, epg	
			Mean ± SD	Maximum
All animals	229	26.9	21 ± 70	518
Dogs	80	35.0	25 ± 73	508
Cats	35	48.6	66 ± 129	518
Pigs	114	14.4	4 ± 18	160

*Epg, eggs per gram feces. †Based on fecal examination.

**Figure 2:**
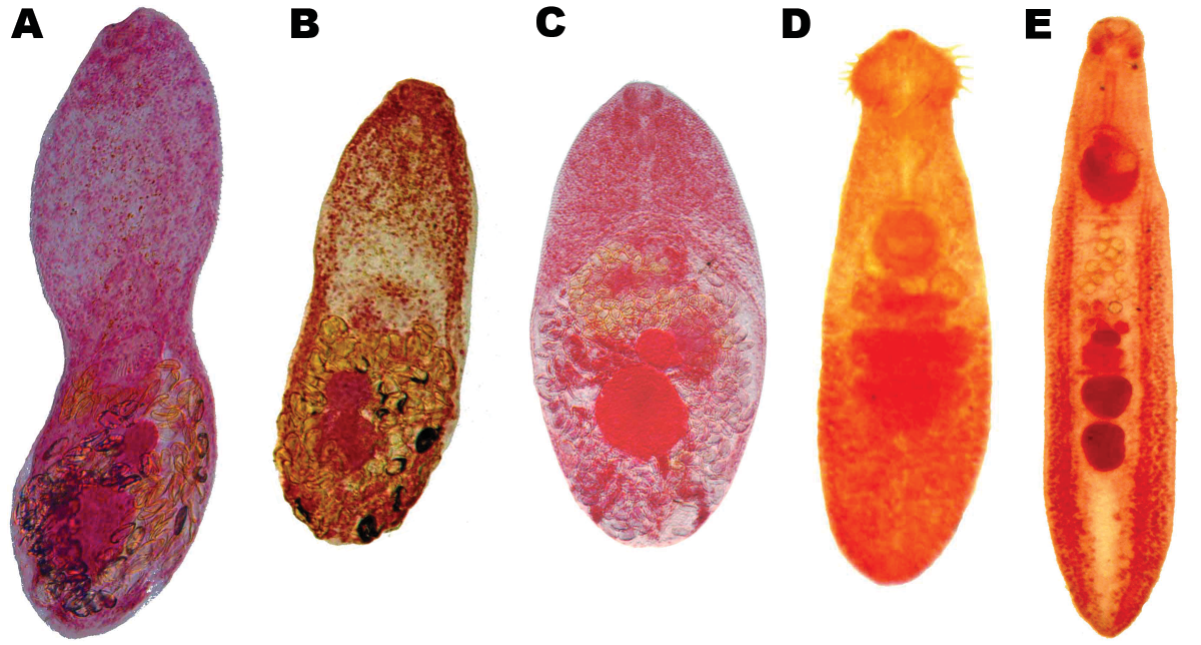
Adult trematodes recovered from domestic animals in Nghe An Province, Vietnam. A) *Haplorchis taichui*; B) *H. pumilio*; C) *H. yokogawai*; D) *Echinochasmus japonicus*; E) *Echinostoma cinetorchis.*

All trematodes recovered from necropsy samples were fishborne-zoonotic intestinal flukes (Table 2)*.* *Haplorchis pumilio* was the most prevalent species in fecal-positive dogs, cats, and pigs, followed by *H. taichui* and *H. yokogawai* (Figure 2).

**Table 2:** Trematode infections in domestic animals, by trematode species, Nghe An Province, Vietnam, November 2005

Animal species	Trematode species	Prevalence, %*	Intensity of adult worms	
			Mean ± SD†	Maximum
Dogs (n = 27)	*Haplorchis pumilio*	92.6	47 ± 83	348
	*H. taichui*	62.9	28 ± 47	160
	*H. yokogawai*	25.9	13 ± 25	69
	*Haplorchis* spp.	62.9	30 ± 41	123
	*Echinochasmus japonicus*	29.6	38 ± 79	231
	*E. perfoliatus*	18.5	3 ± 3	9
	*Echinostoma cinetorchis*	3.7	2	2
	*Centrocestus formosanus*	11.1	16 ± 24	44
	Not identified	11.1	10 ± 8	15
	All	100	99 ± 201	924
Cats (n = 18)	*H. pumilio*	100	33 ± 62	268
	*H. taichui*	77.8	33 ± 94	357
	*H. yokogawai*	11.1	105 ± 136	201
	*Haplorchis* spp.	77.8	51 ± 135	514
	*E. perforliatus*	5.6	3	3
	*C. formosanus*	5.6	4	4
	Not identified	22.2	8 ± 4	10
	All	100	112 ± 309	1,340
Pigs (n = 5)	*H. pumilio*	100	5 ± 4	13
	*H. taichui*	60	2	2
	*H. yokogawai*	40	2	2
	*Haplorchis* spp.	100	12 ± 13	29
	*E. japonicus*	60	3 ± 3	6
	Not identified	60	13 ± 16	32
	All	100	29 ± 32	84

*Based on estimation of worm burdens in animals with positive fecal egg counts. †SD provided only for trematode species in >1 animal.

### Association between Risk Factors and Infections

Distributions of dogs, cats, and pigs were comparable in the 5 districts. According to the questionnaire, 100% of cats and 95.0% of dogs were free roaming, whereas 94.7% of pigs were confined. Feeding raw fish to dogs, cats, and pigs was reported by 41.3%, 45.7%, and 36.8% of farmers, respectively. Raw fish consumption was observed for 61.3% of dogs and 62.8% of cats. Univariable analysis showed the following to be significantly associated with FZT infections: animal species, free roaming, being fed raw fish, eating raw fish, catching fish from canals, and farmers giving dead fish from fish ponds to animals (Table 3).

**Table 3:** Risk factors for fish-borne zoonotic trematodes infection in domestic animals, Nghe An Province, Vietnam, November 2005*

Variable	No. samples	Univariable analysis		
		Crude OR	95% CI	p value
Animal species†				<0.001
Dogs	80	3.3	1.6–6.6	
Cats	35	5.7	2.5–13.5	
Pigs	114	1	–	
Age, mo				
<12	174	1.2	0.6–2.3	0.64
>12	55	1	–	
Free-roaming				0.0001
Always	117	0.6	0.4–0.8	
Sometimes	4	Inf		
Never	108	1	–	
Fed raw fish				
Sometimes	91	1.9	1.0–3.4	0.04
Never	138	1		
Eats raw fish				
Sometimes	158	4.0	2.2–7.5	<0.0001
Never	71	1		

*Data from all dogs, cats, and pigs were analyzed. OR, odds ratio; CI, confidence interval; Inf, infinity. †Multivariable analysis showed an interaction only between animal species and feeding raw fish; p = 0.02.

Goodness-of-fit test for all observations was 1.013, which suggests that the model fits the data. Multivariable logistic analysis showed that, overall, animal species was strongly associated with FZT infections; however, an interaction was found between animal species and being fed raw fish. The risk of being infected was 4.75× higher for pigs fed raw fish than for pigs not fed raw fish. Risk for infection was higher for dogs and cats, regardless of whether they were fed raw fish, than for pigs that were not fed raw fish. Factors not significantly associated with infection in dogs, cats, or pigs were sex, ability to roam freely, district, age, access to fish from canals, being fed dead fish from ponds, deworming, composting of feces, and place of defecation.

Dogs that regularly ate raw fish had a 3.4× higher risk (odds ratio) of being infected than dogs that did not (p = 0.017, 95% confidence interval 1.18–9.74). In contrast, no such significant difference was observed for cats and pigs (data not shown).

### Association between Infections in Domestic Animals and in Cultured Fish

From 48 of the 51 fish-farming households, 89.6% of these households had FZT-infected fish and 68.8% had FZT-infected domestic animals. Fisher exact test showed significant associations between the infections in fish and domestic animals (p = 0.028) and, at the animal species level, between infections in fish and cats (p = 0.042).

### Relative Transmission Index

TDEE from humans and domestic animals in the 5 districts in Nghe An Province was 933 × 10^6^, of which 371 × 10^6^ eggs were from pigs (Table 4). RTI was highest in pigs, followed by dogs and humans, and lowest in cats. Although FZT prevalence was lowest for pigs, their contribution to egg contamination in the community was the greatest.

**Table 4:** Total daily egg excretions and relative transmission index of domestic animals and humans, Nghe An Province, Vietnam, November 2005*

Species	Total no. animals	Prevalence, %	Intensity, epg	TDEE, 10^6^	RTI, %
Humans	886,700	0.6†	215†	183	19
Dogs	332,039	35.0	25	288	31
Cats	141,254	48.6	66	91	10
Pigs	425,306	14.4	4	371	40

*Epg, eggs per gram; TDEE, total daily egg excretions; RTI, relative transmission index. †Data obtained from Olsen et al. (*5*) and Annette Olsen (pers. comm.).

## Discussion

**Figure 3:**
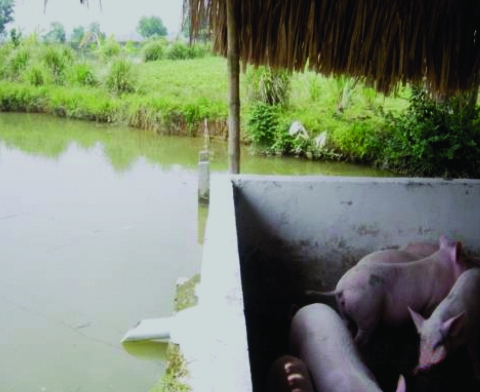
Typical pig pen built on bank of fish pond in Nghe An Province, Vietnam. The design allows fecal waste to drain into the pond.

The likelihood that these reservoir hosts have a major role in sustaining transmission of FZT to cultured fish, regardless of prevalence in humans, is high on the basis of the following: relatively high FZT prevalence, intensity of egg excretion and RTI in domestic animals, and the similar FZT species composition found in infected domestic animals and infected fish. Although prevalence and intensity were markedly lower for pigs, their relatively large amount of feces makes them a major source of FZT eggs that can contaminate local bodies of water and infect snails (Figure 3). Among the other animal species, dogs excreted about 3× more FZT eggs than did cats. We conclude that these domestic animals, especially dogs and pigs, play a major role in the epidemiology of FZT in aquaculture. Although reports from other Southeast Asian countries have suggested that nonhuman reservoir hosts play only a minor role in the epidemiology of liver flukes, specific studies on the role of reservoir hosts, other than ours, have not been carried out (*1*,*13**,**14*). One limitation of our study is that the use of 2 different methods to obtain prevalence data from humans (*5*) and domestic animals may have introduced a bias when comparing the relative contributions of the eggs to the environment. However, the Kato-Katz method and our method were evaluated as reliable and the most suitable methods for detection of eggs in human and domestic animal studies, respectively (*8*,*15*).

The prevalence and species diversity of FZT in dogs, cats, and pigs in Vietnam (*H. pumilio, H. taichui, H. yokogawai, Echinochasmus japonicus, E. perfoliatus, Echinostoma cinetorchis*, and *Centrocestus formosanus*) may represent an emerging problem. Each of these species has been linked to health problems in humans (*1*,*16*). Intestinal trematodes in Thailand and Korea are also often reported (*17**,**18*), and recently, mixed FZT infections in humans in Vietnam and Laos were reported (*3*,*19*). Although the liver fluke *Clonorchis sinensis* was recently reported to have been found in humans in northern Vietnam (*3*), it was not detected in humans or fish in this fish farming area (*5**,**6*)*.* Liver fluke distribution may thus be limited compared with intestinal fluke distribution; however, more geographically comprehensive surveys are needed.

The generally higher prevalence and intensity of infections for dogs and cats than for pigs can be explained by the free roaming of dogs and cats, which allows them greater access to fish from the ponds or pond banks; pigs are normally confined and are only exposed to infection if farmers feed them raw fish or fish waste. This explanation is supported by risk-factor analysis showing that the risk for FZT infections in dogs was related to their behavior of eating raw fish whereas infections in pigs were closely related to their being fed raw fish or fish waste. This finding suggests that educating farmers about preventive animal husbandry practices could affect FZT transmission.

Fish-eating birds and ducks are also known definitive hosts for some FZT species (*20**,**21*) and may contaminate fish ponds with eggs; however, they were excluded from this study because of an avian influenza epidemic in the study areas during sampling. To our knowledge, prevalence of FZT in fish-eating birds and ducks has not been systematically investigated in Southeast Asia, although avian infections are documented (*20*,*21*). The role of fish-eating birds and ducks in the epidemiology of FZT should be assessed.

In conclusion, prevention of FZT infections in domestic animals must be included in any public health strategy to control FZT in humans in fish-farming communities. This can be accomplished by including these hosts in drug-treatment programs aimed at their human owners, proper disposal or inactivation of eggs in feces that may contaminate water, and education of farmers about the dangers of risky feeding practices.

## References

[1] Chai JY, Murrell KD, Lymbery AJ. Fish-borne parasitic zoonoses: status and issues. Int J Parasitol. 2005;35:1233–54. 10.1016/j.ijpara.2005.07.013 16143336

[2] Murrell KD, Fried B. Food-borne parasitic zoonoses, fish and plant-borne parasites. New York: Springer; 2007. p. 3–115.

[3] Trung Dung D, Van De N, Waikagul J, Dalsgaard A, Chai JY, Sohn WM, Fishborne zoonotic intestinal trematodes, Vietnam. Emerg Infect Dis. 2007;13:1828–33. 18258031 10.3201/eid1312.070554 PMC2876759

[4] World Health Organization. Report of joint WHO/FAO workshop on food-borne trematode infections in Asia, Hanoi, Vietnam. Organization. 2004;1–58.

[5] Olsen A, Le KT, Murrell KD, Dalsgaard A, Johansen MV, De NV. Cross-sectional parasitological survey for helminth infections among fish farmers in Nghe An province. Vietnam. Acta Trop. 2006;100:199–204. 10.1016/j.actatropica.2006.10.010 17141724

[6] Chi TTK, Dalsgaard A, Turnbull JF, Pham AT, Murrell KD. Prevalence of zoonotic trematodes in fish from Vietnamese fish-farming community. J Parasitol. 2008;94:423–8. 10.1645/GE-1389.1 18564743

[7] Willingham AL, Johansen MV, Barnes BH. A new technic for counting *Schistosoma japonicum* eggs in pig feces. Southeast Asian J Trop Med Public Health. 1998;29:128–30. 9740285

[8] Anh NT, Phuong NT, Ha GH, Thu LT, Johansen MV, Murrell DK, Evaluation of techniques for detection of small trematode eggs in feces of domestic animals. Vet Parasitol. 2008;156:346–9. 10.1016/j.vetpar.2008.05.021 18583060

[9] Pearson JC, Ow-Yang CK. New species of *Haplorchis* from Southeast Asia, together with keys to the *Haplorchis*-group of heterophyid trematodes of the region. Southeast Asian J Trop Med Public Health. 1982;13:35–60. 7112214

[10] Yamaguti S. Synopsis of digenetic trematodes of vertebrates, vol. 1. Tokyo: Keigaku Publishing Company; 1971.

[11] Wang TP, Johansen MV, Zhang SQ, Wang FF, Wu WD, Zhang GH, Transmission of *Schistosoma japonicum* by humans and domestic animals in the Yangtze River valley, Anhui province, China. Acta Trop. 2005;96:198–204. 10.1016/j.actatropica.2005.07.017 16188215

[12] Atlas VA. Hanoi, Vietnam: Cartographic Publishing House; 2005.

[13] Rim HJ. Clonorchiasis: an update. J Helminthol. 2005;79:269–81. 10.1079/JOH2005300 16153321

[14] Sadun EH. Studies on *Opisthorchis viverrini* in Thailand. Am J Hyg. 1955;62:81–115. 13258561 10.1093/oxfordjournals.aje.a119772

[15] Hong ST, Choi MH, Kim CH, Chung BS, Ji Z. The Kato-Katz method is reliable for diagnosis of *Clonorchis sinensis* infection. Diagn Microbiol Infect Dis. 2003;47:345–7. 10.1016/S0732-8893(03)00113-5 12967748

[16] Chai JY. Intestinal flukes. In: Murrell KD, Fried B, editors. Food-borne parasitic zoonoses. New York: Springer; 2007. p. 53–116.

[17] Hong SJ, Seo BS, Lee SH, Chai JY. A human case of *Centrocestus armatus* infection in Korea. Korean J Parasitol. 1988;26:55–60. 10.3347/kjp.1988.26.1.55 12811033

[18] Chai JY, Lee SH. Food-borne intestinal trematode infections in the Republic of Korea. Parasitol Int. 2002;51:129–54. 10.1016/S1383-5769(02)00008-9 12113752

[19] Chai JY, Han ET, Guk SM, Shin EH, Sohn WM, Yong TS, High prevalence of liver and intestinal fluke infections among residents of Savannakhet Province in Laos. Korean J Parasitol. 2007;45:213–8. 10.3347/kjp.2007.45.3.213 17876167 PMC2526321

[20] Pearson JC. A revision of the subfamily Haplorchinae Loss, 1899 (Trematoda: Heterophyinae). Parasitology. 1964;54:604–76. 10.1017/s003118200008269x 14227629

[21] Eom KS, Rim HJ, Jang DH. A study on the parasitic helminths of domestic duck (*Anas platyrhynchos* var. *domestica Linnaeus*) in Korea. Korean J Parasitol. 1984;22:215–21. 10.3347/kjp.1984.22.2.215 12891015

